# Organometallic *cis*-Dichlorido Ruthenium(II) Ammine Complexes

**DOI:** 10.1002/ejic.201100250

**Published:** 2011-06-22

**Authors:** Soledad Betanzos-Lara, Abraha Habtemariam, Guy J Clarkson, Peter J Sadler

**Affiliations:** [a] Department of Chemistry, University of WarwickCoventry, CV4 7AL, UK E-mail: P.J.Sadler@warwick.ac.uk

**Keywords:** Ruthenium, Sandwich complexes, Cytotoxicity, Antitumor agents, Arene ligands, N ligands

## Abstract

Bifunctional neutral half-sandwich Ru^II^ complexes of the type [(η^6^-arene)Ru(NH_3_)Cl_2_] where arene is *p*-cym (**1**) or bip (**2**) were synthesised by the reaction of *N*,*N*-dimethylbenzylamine (dmba), NH_4_PF_6_ and the corresponding Ru^II^ arene dimer, and were fully characterised. X-ray crystallographic studies of [(η^6^-*p*-cym)Ru(NH_3_)Cl_2_]**·**{(dmba–H)(PF_6_)} (**1a**) and [(η^6^-bip)Ru(NH_3_)Cl_2_] (**2**) show extensive H-bond interactions in the solid state, mainly involving the NH_3_ and the Cl ligands, as well as weak aromatic stacking interactions. The half-lives for the sequential hydrolysis of **1** and **2** determined by UV/Vis spectroscopy at 310 K ranged from a few minutes for the first aquation to ca. 45 min for the second aquation; the diaqua adducts were the predominant species at equilibrium. Arene loss during the aquation of complex **2** was observed. Upon hydrolysis, both complexes readily formed mono- and di-9-ethylguanine (9-EtG) adducts in aqueous solution at 310 K. The reaction reached equilibrium after ca. 1.8 h in the case of complex **1** and was slower but more complete for complex **2** (before the onset of arene loss at ca. 2.7 h). Complexes **1** and **2** were not cytotoxic towards A2780 human ovarian cancer cells up to the maximum concentration tested (100 μM).

## Introduction

**Figure 1:**
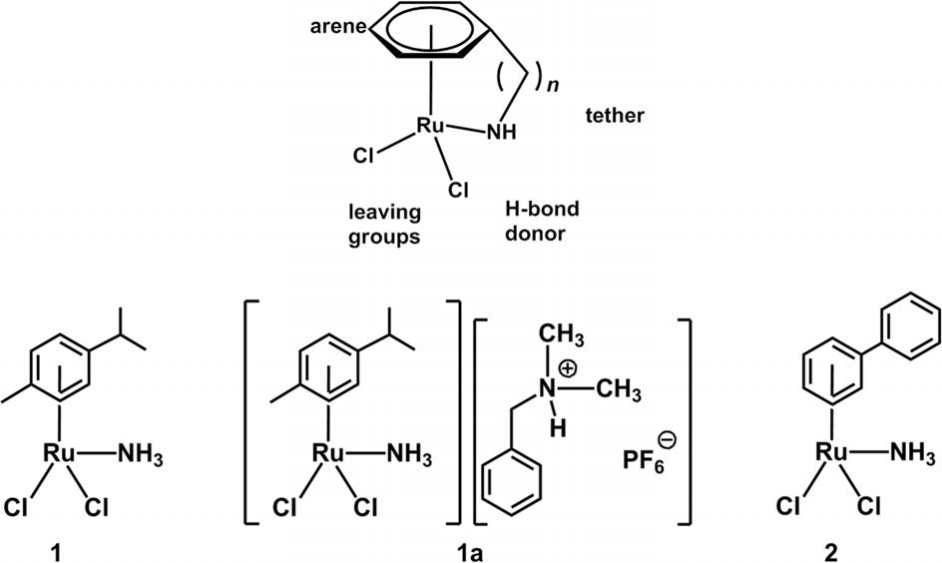
Top: General structure of amine-tethered dichlorido Ru^II^ arene complexes. Bottom: Structures of the neutral Ru^II^ arene complexes [(η^6^-*p*-cym)Ru(NH_3_)Cl_2_] (**1**), [(η^6^-*p*-cym)Ru(NH_3_)Cl_2_]**·**{(dmba–H)(PF_6_)} (**1a**) and [(η^6^-bip)Ru(NH_3_)Cl_2_] (**2**) studied in this work.

Soon after the discovery of the cytotoxic properties of cisplatin,^1^–^3^ extensive studies of platinum am(m)ine halido analogues led to a series of empirical rules governing the chemotherapeutic potential of this class of derivatives.^4^ It was concluded that active compounds should: (i) be neutral, presumably to facilitate passive diffusion into cells; (ii) have two leaving groups in a *cis*-configuration; (iii) contain non-leaving groups with poor *trans*-labilising ability, similar to that of NH_3_ or organic amines; and (iv) have leaving groups with a window of lability centred on the chlorido ligand. Recent studies have focused on applying analogous structure-activity relationships to other metal complexes for anticancer drug design.^5^ Half-sandwich Ru^II^ arene complexes of the general formula [(η^6^-arene)Ru(X)(Y)(Z)]^*n*+^ where X and Y are two monodentate ligands or if linked a bidentate chelating ligand, and Z is a leaving group, have recently been shown to have potential as anticancer drugs.^6^ In contrast, bifunctional neutral dichlorido Ru^II^ agents containing tethered ligands of general formula [(η^6^:η^1^-arene:N)RuCl_2_], Figure 1, as well as the more stable derivatives [(η^6^:η^1^-arene:N)Ru(oxalate)],^7^,^8^ have proved to be inactive. The first reported synthesis and structural characterisation of an organometallic Ru^II^ complex with similarity to cisplatin, bearing two ammonia ligands and one chlorido ligand, [(η^6^-benzene)Ru(NH_3_)_2_Cl][PF_6_],^9^,^10^ appeared thirty years ago and was studied for its chemical properties. More recently, the synthesis and properties of the *p*-cymene complex [(η^6^-*p*-cym)Ru(NH_3_)_2_Cl]^+^ have been described.^11^ This complex is much less potent than cisplatin, displaying an IC_50_ value (50 % inhibitory concentration) 500 times larger than that for the platinum drug under the same conditions. This lack of activity has been attributed to its instability both in aqueous media and organic solutions. The synthesis of numerous dichlorido Ru^II^ arene complexes bearing N-donor ligands^12^ (mainly, but not restricted to, pyridine derivatives) and mixed donor ligands (such as phosphanes)^13^ has also been reported. These dichlorido complexes not only display interesting biological activities,^14^ but have also found use in other applications, such as catalysts for organic synthesis.^15^,^16^ The synthesis of bifunctional Ru^II^ arene complexes has also included the use of bulkier N-monodentate ligands such as paullone derivatives,^17^ and pyr(id)ones,^18^ or non-N-based monodentate ligands such as 1,3,5-triaza-7-phosphatricyclo[3.3.1.1]decane (pta),^19^ as well as biologically active groups such as staurosporine^20^ or tyrphostin^21^ derivatives. Moreover, based on several studies with platinum anticancer compounds,^22^,^39^ it seems that the presence of an H-bond donor atom may be a desirable feature in the design of bifunctional Ru^II^ arene complexes in order to, for example, stabilise intrastrand cross-linking on DNA in a similar fashion to cisplatin.^23^ For this, monodentate NH_2_R ligands have been used.^24^,^25^ However, the evidence suggests that the coordinated N-donor group can undergo facile ligand-substitution reactions in solution and complexes such as [(η^6^-mesitylene)Ru(NHR_2_)Cl_2_] where R is ethyl or butyl, readily decompose in solution.^26^ In the work reported here, the synthesis and structural characterisation of the first examples of neutral water-soluble organometallic Ru^II^ complexes which possess an ammonia ligand and two *cis* chlorido ligands, are described. Their aqueous solution chemistry, ability to bind to model DNA nucleobases, and cytotoxicity towards human cancer cells were also investigated.

## Results and Discussion

### Synthesis

Two new Ru^II^ arene complexes [(η^6^-arene)Ru(NH_3_)Cl_2_] where arene is *p*-cym (**1**) or bip (**2**), showing constitutional similarity to cisplatin were synthesised in good yields (68 % and 66 %, respectively), Figure 1. The first report of the synthesis of organometallic complexes containing ammonia and chlorido ligands was thirty years ago^10^ and resulted in the monochlorido bisammine complexes [(η^6^-benzene)M(NH_3_)_2_Cl][PF_6_] (where M = Ru or Os). The synthetic routes were rather complicated and often gave mixtures of products. They involved reacting [(η^6^-benzene)M(Cl)_2_]_2_ dimers in concentrated aqueous ammonia and methanol, followed by the addition of a saturated aqueous NH_4_PF_6_ solution to precipitate the complexes as the corresponding PF_6_ salts. In the present work, the reaction of [(η^6^-arene)RuCl_2_]_2_ where the arene is *p*-cym or bip, with 2 mol equiv. of *N*,*N*-dimethylbenzylamine (dmba) and 2 mol equivalents of NH_4_PF_6_ in dry MeOH under a N_2_ atmosphere at ambient temperature proved to be convenient. The first step in the synthesis is believed to involve a fast reaction between the base *N*,*N*-dimethylbenzylamine (dmba) and NH_4_^+^ to form NH_3_ in situ which then reacts with the Ru^II^ arene dimer to afford complexes **1** and **2**. A similar reaction mechanism has also been suggested for the synthesis of the cationic complex [(η^6^-*p*-cym)Ru(NH_3_)_2_Cl]^+^.^11^ During the purification steps to afford complex **1**, the presence of the *N*,*N*-dimethylbenzylammonium ion (dmba–H)^+^ was detected in solution by ^1^H NMR spectroscopy prior to recrystallisation of the reaction product. The signals were assigned to the adduct [(η^6^-*p*-cym)Ru(NH_3_)Cl_2_]**·**{(dmba–H)(PF_6_)} (**1a**) [(CD_3_)_2_CO, 500 MHz *δ*_H_: 3.01 (s, 6 H), 4.50 (s, 2 H), 7.50 (m, 3 H), 7.66 ppm (m, 2 H)], thus providing further evidence to support the proposed mechanism. Furthermore, the nature of the solvent appears to play an important role in the quality of the products; syntheses carried out in CH_3_CN or CH_2_Cl_2_ also yielded complexes **1** and **2** but in lower yields (less than 40 % in both cases). This observation suggests that the reaction might proceed via a nucleophilic substitution pathway. The intermediate species generated upon the nucleophilic attack of NH_3_ on the Ru^II^ centre might be considerably better stabilized by interactions with a solvent of medium polarity-index such as methanol (5.1), compared to either a more polar solvent as CH_3_CN (5.8) or less polar solvent such as CH_2_Cl_2_ (3.1).^27^

### Characterisation

**Figure 2:**
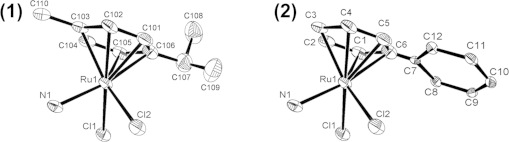
X-ray crystal structures of [(η^6^-*p*-cym)Ru(NH_3_)Cl_2_]**·**{(dmba–H)(PF_6_)} (**1a**) and [(η^6^-bip)Ru(NH_3_)Cl_2_] (**2**). Thermal ellipsoids show 50 % probability. The hydrogen atoms have been omitted for clarity. The ion pair in **1a** is not shown.

**Figure 3:**
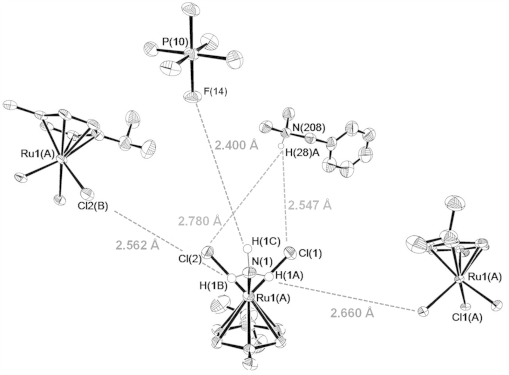
Intermolecular H-bond interactions present in the X-ray crystal structure of [(η^6^-*p*-cym)Ru(NH_3_)Cl_2_]**·**{(dmba–H)(PF_6_)} (**1a**). Atoms not involved in the specified interactions are omitted for clarity.

**Figure 4:**
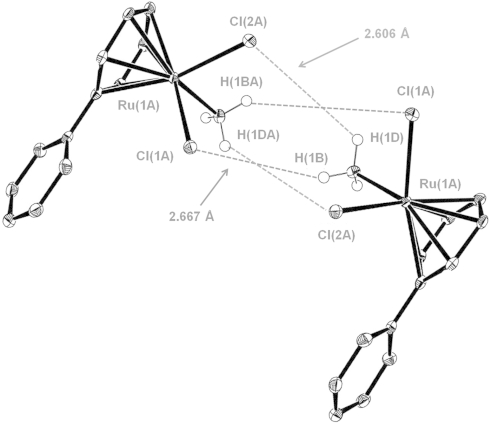
X-ray crystal structure of [(η^6^-bip)Ru(NH_3_)Cl_2_] (**2**) showing N–H**···**Cl contacts.

**Figure 5:**
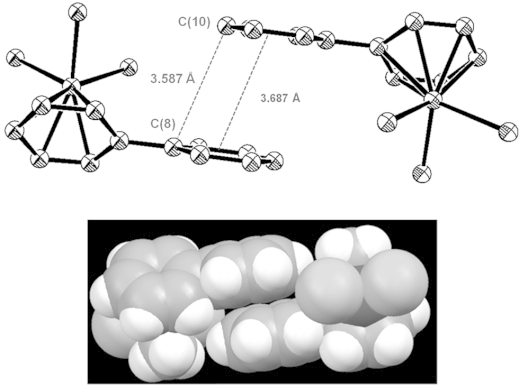
X-ray crystal structure of [(η^6^-bip)Ru(NH_3_)Cl_2_] (**2**) showing a π-π stacking interaction between two uncoordinated bip rings in neighbouring molecules. Inset: space-filling model.

Both complexes were fully characterised by 1D and 2D ^1^H NMR methods as well as elemental analysis, ESI-MS, and X-ray crystallography. The ^1^H NMR resonances of both arenes in complexes **1** and **2** are high-field-shifted by about 1 ppm compared to the corresponding parent dimers. The resonances for the NH protons in both complexes can be readily assigned in a non-aqueous deuterated solvent such as acetone. Figure S1 shows the ^1^H NMR spectrum of complex [(η^6^-*p*-cym)Ru(NH_3_)Cl_2_] (**1**) in [D_6_]acetone as an example. Suitable crystals of [(η^6^-*p*-cym)Ru(NH_3_)Cl_2_]**·**{(dmba–H)(PF_6_)} (**1a**) were grown using the crude product isolated from the synthesis (and prior to recrystallisation) in a saturated dichloromethane solution at ambient temperature, whereas crystals of the biphenyl complex [(η^6^-bip)Ru(NH_3_)Cl_2_] (**2**) were obtained from a saturated acetonitrile solution at ambient temperature. Selected bond lengths and angles are given in Table 1. The structures and atom labelling are shown in Figure 2 and the crystallographic data are listed in Table S1. The two neutral Ru^II^ arene complexes adopt the familiar pseudo-octahedral three-legged piano stool geometry common to other half-sandwich Ru^II^ arene structures.^6^,^28^ The Ru atom is η^6^-bonded to *p*-cym in **1a** and bip in **2**, coordinated to an ammonia nitrogen, and to two chloride ions which constitute the three legs of the piano stool. The unit cell of the Ru^II^ arene complex **1a** also contains the *N*,*N*-dimethylbenzylammonium hexafluorophosphate ionic pair (dmba–H)(PF_6_). The Ru–Cl bonds in complexes **1a** [2.421(2)/2.427(2) Å] and **2** [2.4246(9)/2.4284(8) Å] as well as the corresponding Ru–N bonds [2.116(8) for **1a** and 2.135(3) Å for **2**] do not vary with a change in arene. The Ru–Cl bond lengths are within the range found for other Ru–N(sp^3^) arene complexes^29^ such as [(η^6^:η^1^-C_6_H_5_(CH_2_)_3_NH_2_)RuCl_2_]^7^ and longer than those found in related structures where the N atom belongs to an aromatic pyridine ring.^30^,^13^ The Ru–N distances are not significantly affected by a change of arene from *p*-cym (**1a**) to bip (**2**) and are within the range found in similar complexes such as [(η^6^-*p*-cym)Ru(NH_3_)_2_Cl]^+^ [2.1504(15) and 2.1425(15) Å].^11^ They are again on average, slightly longer than those of analogous complexes where the nitrogen donor atom belongs to an aromatic system such as a pyridine derivative.^11^ The Ru**···***p*-cym_(centroid)_ distance (as measured from Mercury version 2.2.) in complex **1a** (1.657 Å) is slightly shorter than that for the bip analogue **2** (1.670 Å). These two distances differ from those observed when the N atom belongs to a (tethered) ethylenediamine^7^,^8^,^32^ (ca. 1.65 Å) but are slightly shorter (ca. 0.02–0.05 Å) than those found in related complexes where the Ru^II^ centre is bound to an aromatic XY-chelating ligand (XY are N, O, or S).^31^ The X-ray crystal structures of compounds **1a** and **2** also show intra and/or intermolecular π-π stacking interactions, a common feature in related Ru^II^ complexes containing extended aromatic rings^32^ as well as H-bond interactions, Figure 3 and Figure 4. Tables S2 and S3 list the hydrogen bond lengths [Å] and angles [°] in the X-ray crystal structures of [(η^6^-*p*-cym)Ru(NH_3_)Cl_2_]**·**{(dmba–H)(PF_6_)} (**1a**) and [(η^6^-bip)Ru(NH_3_)Cl_2_] (**2**), respectively. The ionic pair in the crystal structure of **1a** also shows π-π stacking interactions, Figure S2. Complex [(η^6^-bip)Ru(NH_3_)Cl_2_] (**2**) pairs with an adjacent molecule also via intermolecular π-π stacking interaction. The mean planes involving the uncoordinated phenyl (ph) rings in the bip arene are parallel, Figure 5. A space-filling model of complex **2** (inset in Figure 5) shows the presence of aromatic stacking with the shortest atomic contact C(8)**···**C(10) being 3.587 Å and a ph_(centroid)_–ph_(centroid)_ distance of 3.687 Å, typical of such weak interactions.^33^ The uncoordinated phenyl ring is tilted by 41.06° relative to the main plane defined by the coordinated ph_(bound)_.

**Table 1:** Selected bond lengths [Å] and angles (°) for [(η^6^-*p*-cym)Ru(NH_3_)Cl_2_]·{(dmba–H)(PF_6_)} (1a) and [(η^6^-bip)Ru(NH_3_)Cl_2_] (2)

Bond length/angle	1a	2
Ru(1)–arene_(centroid)_^[a]^	1.657	1.670
Ru(1)–Cl(1)	2.427(2)	2.4284(8)
Ru(1)–Cl(2)	2.421(2)	2.4246(9)
Ru(1)–N(1)	2.116(8)	2.135(3)
N(1)–Ru(1)–Cl(1)	83.80(2)	84.29(8)
N(1)–Ru(1)–Cl(2)	84.30(2)	84.73(8)
Cl(2)–Ru(1)–Cl(1)	85.11(8)	85.26(3)

[a] Calculated with Mercury, version 2.2.

### Kinetics of Hydrolysis

**Figure 6:**
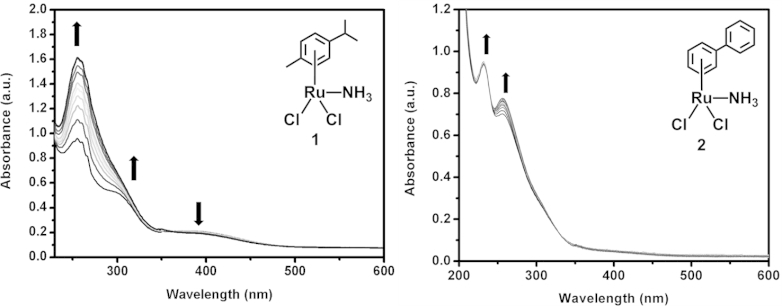
Time evolution of the hydrolysis reaction for 100 μm (5 % MeOH/95 % H_2_O) solutions of complexes **1** and **2** at 310 K followed by UV/Vis spectroscopy.

Dissolution of compounds **1** and **2** in 5 % MeOH/95 % H_2_O (100 μm) at 310 K gave rise to ligand-substitution reactions at the Ru^II^ centre as indicated by rapid changes in UV/Vis absorption bands. The time evolution spectra for the two Ru^II^ arene complexes at 310 K are shown in Figure 6. The initial electronic absorption spectrum of aqueous [(η^6^-*p*-cym)Ru(NH_3_)Cl_2_] (**1**) at 310 K exhibits peaks with maxima at ca. 255, 320, and 410 nm. The highest energy absorption band increases in intensity while the lower energy band decreases. The band centred at ca. 320 nm disappears upon hydrolysis. In the case of [(η^6^-bip)Ru(NH_3_)Cl_2_] (**2**) in aqueous solution, the initial electronic absorption spectrum at 310 K exhibits peaks with maxima at ca. 255 and 274 nm which increased in intensity only slightly over a period of ca. 6 h. Related mononuclear Ru^II^ arene complexes of the type [(η^6^-arene)Ru(X)(Y)Cl]^*n*+^ where XY is a bidentate chelating ligand^34^–^36^ are known to undergo a monoexponential decrease in absorbance due to the loss of one chloride and substitution by water.^37^ The presence of two chlorido ligands that can be substituted by water in complexes **1** and **2** and the absence of a clear isosbestic point in their UV/Vis absorption spectra correlates with a bi-exponential kinetics as shown by the fit to the data, Figure S3. The rate constants for hydrolysis of complex **2** were determined over a 160 min period.

The hydrolysis rate constants and half-lives are listed in Table 2. It can be seen that the first step is fast for both complexes (half-lives ca. 1 min). The aquation rates for the second step are also similar for both complexes. The loss of the first chlorido ligand might be expected to be faster than the substitution of the second because of the increase in positive charge on the molecule. This is the case for both complexes **1** and **2**; the first hydrolysis rate (*k*_1_) of complex **1** is not only ca. 48 times faster than the second (*k*_2_) but also ca. 1.5 times faster than *k*_1_ for the bip complex **2**.

**Table 2:** Hydrolysis data for complexes 1 and 2 (100 μm in 5 % MeOH/95 % H_2_O) at 310 K determined by UV/Vis spectroscopy

	1	2

First aquation		
*k*_1_ × 10^–3^ [min^–1^]^[a,b]^	720.0 ± 21.8	555.0 ± 27.2
*t*_1/2_ [min]	1.0	1.2

Second aquation		
*k*_2_ × 10^–3^ [min^–1^]^[a,b]^	15.2 ± 0.7	15.9 ± 5.8
*t*_1/2_ [min]	45.7	43.6

[a] The errors quoted are fitting errors. [b] The rate constants for complex **2** were determined over the period of time before the onset of arene loss (at ca. 160 min detected by ^1^H NMR).

### Hydrolysis Equilibria

Upon dissolution of complexes **1** or **2** in D_2_O, the ^1^H NMR spectrum shows the presence of a number of species. In order to characterise the products of hydrolysis and determine the extent of the reactions, freshly-made 100 μm solutions of complexes **1** and **2** (in 5 % [D_4_]MeOD/95 % D_2_O) were allowed to equilibrate for 24 h at 310 K and were then studied at the same temperature using ^1^H NMR spectroscopy. Figure S4 shows the time dependence of the ^1^H NMR CH_3(isopropyl)_ peaks of the *p*-cym ring of complex **1** at 310 K. The ^1^H NMR spectra of complexes **1** and **2** initially contained one major set of peaks (dichlorido species) and then a second and third set of peaks, which increased in intensity over time. The new sets of peaks were assigned to the mono- and di-aqua adducts, [(η^6^-arene)Ru(NH_3_)(OH_2_)Cl]^+^ and [(η^6^-arene)Ru(NH_3_)(OH_2_)_2_]^2+^, respectively. The mass-to-charge ratios of peaks in ESI-MS spectra of the solutions after 24 h were consistent with the formation of the di-aqua complexes; Table S4 (observed as the base peak in the spectra). For complex [(η^6^-bip)Ru(NH_3_)Cl_2_] (**2**) an additional set of ^1^H NMR peaks [*δ* = 7.88 (d), 7.70 (t), 7.42 (t)] assignable to free bip was also observed after 160 min.

Table 3 summarizes the percentage of species detected at equilibrium and after 24 h of reaction as determined by ^1^H NMR peak integration. These data suggest that the *p*-cym complex **1** undergoes mono- and di-hydrolysis reactions to a larger extent (ca. 2 to 7 times) than complex **2** (before biphenyl loss). Changing the arene from *p*-cym (**1**) to bip (**2**) greatly reduces the stability of the complex in aqueous solution at 310 K since no loss of the arene *p*-cym was observed for complex **1**.

**Table 3:** Percentage of species present at equilibrium in 100 μm solutions of complexes 1 and 2 (5 % [D_4_]MeOD/95 % D_2_O) after 24 h at 310 K determined by ^1^H NMR spectroscopy

	% Species			
	Ru–Cl_2_	Ru–OH_2_	Ru–(OH_2_)_2_	Arene loss
[(η^6^-*p*-cym)Ru(NH_3_)Cl_2_] (**1**)	3.1	20.8	76.1	0.0
[(η^6^-bip)Ru(NH_3_)Cl_2_] (**2**)	24^[a]^	2^[a]^	18^[a]^	56^[a]^

[a] Approximate values due to peak overlap.

### Interactions with Nucleobases

DNA is one of the potential targets for transition metal anticancer complexes.^38^ For this reason, reactions of complexes **1** and **2** with 9-ethylguanine (9-EtG) as a model nucleobase were investigated. The interactions were studied by multidimensional ^1^H NMR spectroscopy and the nature of the products verified by ESI-MS. 9-EtG (2 mol-equiv.) was added to an NMR tube containing a 100 μm solution of **1** or **2** in 5 % [D_4_]MeOD/95 % D_2_O at 310 K and the reaction was then followed for ca. 6 h. The interactions occur via the initial in situ formation of the corresponding reactive mono- and di-aqua adducts for each complex. After ca. 10 min of reaction, a second and third set of low-field-shifted peaks appeared and increased in intensity. These peaks are tentatively assigned to the corresponding mono- and dinucleobase adducts with coordination of the Ru^II^ centre to *N7* of 9-EtG. This coordination mode has been previously observed for similar Ru^II^ arene guanine and adenine adducts^39^,^40^,36a and is confirmed by the changes in chemical shift of the H8 peak for bound 9-EtG-*N7* in the monoguanine Ru^II^ arene adducts of both complexes [(η^6^-*p*-cym)Ru(NH_3_)(9-EtG-*N7*)Cl]^+^ (**1-EtG**) or [(η^6^-bip)Ru(NH_3_)(9-EtG-*N7*)Cl]^+^ (**2-EtG**). These have chemical shifts of ca. 8.20 ppm, cf. 8.08 ppm for free 9-EtG under the same conditions. Metallation at the *N7* site of purine bases usually produces a low field shift of the H8 resonance by about 0.3–1.0 ppm,^41^,^42^ The second singlet was assigned to the corresponding diguanine adducts [(η^6^-*p*-cym)Ru(NH_3_)(9-EtG-*N7*)_2_]^2+^ (**1-EtG_2_**) or [(η^6^-bip)Ru(NH_3_)(9-EtG-*N7*)_2_]^2+^ (**2-EtG_2_**) and has a chemical shift in the range of 8.30–8.40 ppm, Figure S5. A similar profile of bifunctional reactivity is well-known for cisplatin, which can form intra- and/or inter-strand crosslinks.^43^–^45^ Furthermore, coordination to two guanine bases has been demonstrated for the fragment {(η^6^-benzene)Ru}^2+^.^46^ Both complexes reacted relatively rapidly with 9-EtG (less than 2 h to reach equilibrium); Table 4 lists the percentage of species detected after 24 h of reaction as determined from integration of ^1^H NMR signals. As it can be seen, at equilibrium complex **1** had reacted with 9-EtG to form ca. 21 % of the corresponding monoguanine adduct [(η^6^-*p*-cym)Ru(NH_3_)(9-EtG-*N7*)Cl]^+^ (**1-EtG**) and 53 % of the diguanine adduct [(η^6^-*p*-cym)Ru(NH_3_)(9-EtG-*N7*)_2_]^2+^ (**1-EtG_2_**). In the case of complex **2**, at equilibrium (before the onset of arene loss, ca. 160 min), ca. 5 % of the corresponding mono 9-EtG adduct [(η^6^-bip)Ru(NH_3_)(9-EtG-*N7*)Cl]^+^ (**2-EtG**) and 32 % of the diguanine adduct [(η^6^-bip)Ru(NH_3_)(9-EtG-*N7*)_2_]^2+^ (**2-EtG_2_**) had been formed. The reactions of complex **2** with 9-EtG take longer to reach equilibrium compared to those of complex **1**, and are less thermodynamically favoured. The mass-to-charge ratios obtained from ESI-MS data were used to characterise some of the guanine adducts (see Table S5).

**Table 4:** Extent of formation of 9-EtG-*N7* adducts on reactions of complexes 1 and 2 with 2 mol equiv. of 9-EtG in 5 % [D_4_]MeOD/95 % D_2_O at 310 K as determined by integration of ^1^H NMR peaks

Compound	Time	% Mono-	% Di-
	[min]	(9-EtG-*N*^7^)	(9-EtG-*N*^7^)
		adduct	adduct
[(η^6^-*p*-cym)Ru(NH_3_)Cl_2_] (**1**)	106^[a]^	20.4	53.0
	1440	20.4	53.0
[(η^6^-bip)Ru(NH_3_)Cl_2_] (**2**)	160^[b]^	5^[b]^	32^[b]^

[a] Time to reach equilibrium. [b] Time before the onset of arene loss; approximate value due to peak overlap.

### Cancer Cell Growth Inhibition

Neither complex **1** nor **2** was cytotoxic towards the A2780 human ovarian cancer cell line up to the maximum concentration tested (100 μm), Table S6. It is known that metal coordination complexes can undergo ligand-substitution reactions with components of the media in which they are dissolved.^47^ In the case of complexes **1** and **2**, the diminished anticancer activity could be due to inactivation by reaction with components of the cell culture media even before reaching the cell or by other biomolecules once within the cell. This same hypothesis of inactivation has been previously suggested for complexes of the type [(η^6^-*p*-cym)Ru(X)(Y)Z] where X, Y or Z are monodentate ligands such as halides, acetonitrile or isonicotinamide.^6^

## Conclusions

We have shown that neutral Ru^II^ half-sandwich complexes containing two *cis* chlorido ligands and an ammine ligand can be synthesised by the reaction of *N*,*N*-dimethylbenzylamine and NH_4_PF_6_, first forming NH_3_ in situ which then reacts with the appropriate Ru^II^ arene dimer to afford complexes [(η^6^-arene)Ru(NH_3_)Cl_2_] **1** (*p*-cym) and **2** (bip). A notable feature of these species in the solid state is the extensive network of H-bond interactions through the NH_3_ and the Cl ligands as well as π-π stacking aromatic interactions, particularly in the case of complex **2**. Hydrolysis of the complexes was relatively rapid and occurred in two steps with half-lives of ca. 1 min and 46 min. Hydrolysis of the bip complex **2** appeared to be followed by arene loss with time. The possibility that DNA could be a target for these complexes in cancer cells was investigated by exploring their reactions with the model DNA nucleobase 9-ethylguanine (9-EtG) in aqueous solution. Both complexes readily formed mono- and diguanine adducts upon hydrolysis. The reaction of complex **1** with 9-EtG was faster, and to a greater extent than that of **2**. Hydrolysis is known to be a mechanism for activation of cytotoxic chlorido metal complexes.^6^ However, none of the neutral complexes were cytotoxic against the A2780 human ovarian cancer cell line up to the maximum concentration tested (100 μm), perhaps because they are too reactive towards components of the cell culture medium, and in the case of **2** due to arene loss.

## Experimental Section

**Materials:** RuCl_3_**·**3H_2_O was purchased from Precious Metals Online (PMO Pty Ltd.) and used as received. *N*,*N*-dimethylbenzylamine (dmba) and NH_4_PF_6_ were obtained from Aldrich. The Ru^II^ arene precursor dimers [(η^6^-*p*-cym)RuCl_2_]_2_ and [(η^6^-bip)RuCl_2_]_2_ where arene is *p*-cymene (*p*-cym) or biphenyl (bip), were prepared following literature methods.^48^ The solvents used for UV/Vis absorption spectroscopy, dry methanol (reagent grade), and for NMR spectroscopy, [D_6_]acetone, [D]chloroform, [D_4_]methanol and D_2_O, were purchased from Aldrich.

**Synthesis of Ruthenium Complexes:** The neutral complexes [(η^6^-arene)Ru(NH_3_)Cl_2_] where arene is *p*-cym or bip were synthesised using a similar procedure. A suspension of the appropriate Ru^II^ arene dimer [(η^6^-arene)RuCl_2_]_2_, *N*,*N*-dimethylbenzylamine (dmba) and NH_4_PF_6_ in 10 mL of dry MeOH was stirred at ambient temperature under N_2_ atmosphere for 18 h. After evaporation of the clear orange solution that formed, the resulting solid was recrystallised by redissolution in the minimum amount of MeOH, CH_2_Cl_2_ or CH_3_CN and leaving to stand at 298 K for 5 h. The precipitate that formed was filtered off and washed with portions of Et_2_O/MeOH and dried overnight in vacuo resulting in a microcrystalline product. Details of the amounts of reactants, colour changes, and nature of the products for the individual reactions are described below, as well as any variations in the synthetic procedure.

**[(η^6^-*p*-cym)Ru(NH_3_)Cl_2_] (1) and [(η^6^-*p*-cym)Ru(NH_3_)Cl_2_]·{(dmba–H)(PF_6_)} (1a):** A solution of [(η^6^-*p*-cym)RuCl_2_]_2_ (0.10 g, 0.16 mmol), *N*,*N*-dimethylbenzylamine (0.05 mL, 0.32 mmol) and NH_4_PF_6_ (0.05 g, 0.32 mmol) in dry MeOH turned from brick red to orange; an orange solid of [(η^6^-*p*-cym)Ru(NH_3_)Cl_2_] (**1**) was obtained by recrystallization from methanol, whereas bright orange crystals suitable for X-ray diffraction of [(η^6^-*p*-cym)Ru(NH_3_)Cl_2_]**·**[(dmba–H)(PF_6_)] (**1a**) were grown from a saturated dichloromethane solution of the crude product at ambient temperature; yield (for recrystallised **1**) 68 % (0.04 g, 0.12 mmol).

**1:** C_10_H_17_Cl_2_NRu (323.23): calcd. 37.16, H 5.30, N 4.33; found C 37.70, H 5.04, N 4.31.

**1a:** C_19_H_31_Cl_2_F_6_N_2_PRu: calcd. C 37.76, H 5.17, N 4.63; found C 37.72, H 5.04, N 4.21. ESI-MS calcd. for C_10_H_18_Cl_2_NRu {[M] + [H^+^]}^+^ *m*/*z* 324.2; found *m*/*z* 324.0. ^1^H NMR for (**1**) [(CD_3_)_2_CO, 500 MHz]: *δ* = 1.30 (d; *J* = 7.50 Hz, 6 H), 2. 20 (s, 3 H), 3.10 (m, 3 H), 5.36 (d, *J* = 6.25 Hz, 2 H), 5.58 (dd, *J* = 6.25 Hz, 2 H) ppm.

**[(η^6^-bip)Ru(NH_3_)Cl_2_] (2):** A solution of [(η^6^-bip)RuCl_2_]_2_ (0.10 g, 0.16 mmol), *N*,*N*-dimethylbenzylamine (dmba) (0.049 mL, 0.32 mmol) and NH_4_PF_6_ (0.05 g, 0.32 mmol) in dry MeOH turned from brick red to orange; a bright orange solid was obtained; yield 66 % (0.04 g, 0.11 mmol). Crystals suitable for X-ray diffraction were grown from an acetonitrile-saturated solution of recrystallised **2** at ambient temperature. C_12_H_13_Cl_2_NRu**·**H_2_O: calcd. C 39.90, H 4.19, N 3.88; found C 39.83, H 4.32, N 4.43. ESI-MS calcd. for C_12_H_14_Cl_2_NRu {[M] + [H^+^]}^+^ *m*/*z* 344.2; found *m*/*z* 343.9. ^1^H NMR [(CD_3_)_2_CO, 500 MHz]: *δ* = 3.39 (m, 3 H), 5.77 (t, *J* = 5.63 Hz, 1 H), 5.93 (t, *J* = 5.63 Hz, 2 H), 6.03 (t, *J* = 5.63 Hz, 2 H), 6.12 (d, *J* = 6.25 Hz, 2 H), 6.24 (d, *J* = 6.25 Hz, 1 H) ppm.

**X-ray Crystallography:** Diffraction data were collected either on an Oxford Diffraction Gemini four-circle system with a Ruby CCD area detector or on a Siemens SMART three-circle system with CCD area detector equipped with an Oxford Cryosystem Cryostream Cooler. All structures were refined by full-matrix least-squares against *F^2^* using SHELXL 97^49^ and were solved by direct methods using SHELXS^50^ (TREF) with additional light atoms found by Fourier methods. Hydrogen atoms were added at calculated positions and refined using a riding model, except the hydrogens on the NH nitrogens which were located in a difference map. Their positions were allowed to refine but with a distance restraint. Anisotropic displacement parameters were used for all non-H atoms; H-atoms were given an isotropic displacement parameter equal to 1.2 (or 1.5 for methyl and NH H-atoms) times the equivalent isotropic displacement parameter of the atom to which they are attached.

CCDC-CCDC-809024 http://www.ccdc.cam.ac.uk/cgi-bin/catreq.cgi(for **1a**) and -CCDC-809025 http://www.ccdc.cam.ac.uk/cgi-bin/catreq.cgi(for **2**) contain the supplementary crystallographic data for this paper. These data can be obtained free of charge from The Cambridge Crystallographic Data Centre via www.ccdc.cam.ac.uk/data_request/cif.

**NMR Spectroscopy:** ^1^H and ^13^C NMR spectra were acquired in 5 mm NMR tubes at 298 K (unless otherwise stated) on a Bruker DRX-500 NMR spectrometer. All data processing was carried out using XWIN NMR version 3.6 (Bruker U. K. Ltd.). ^1^H NMR chemical shifts were internally referenced to TMS via 1,4-dioxane in D_2_O (*δ* = 3.71 ppm) or residual CHCl_3_ (*δ* = 7.27 ppm), CD_2_HOD (*δ* = 3.31 ppm) or DMSO (*δ* = 2.50 ppm). 1D spectra were recorded using standard pulse sequences. Typically, data were acquired with 128 transients into 16 k data points over a spectral width of 14 ppm. 2D spectra were recorded using standard pulse-pulse sequences. COSY was used to identify pairs of nuclei which are *J*-coupled to one another. Typically, data were acquired with 72 transients into 1024 k data points over a spectral width of 14 ppm (unless otherwise stated) using a relaxation delay of 1.5 s and a mixing time of 0.06 s.

**Elemental Analysis:** Elemental analyses were performed by the Warwick Analytical Service which is the analytical division of Exeter Analytical (U. K. Ltd.) using an Exeter Analytical Elemental Analyzer (CE440).

**Electrospray Ionization Mass Spectrometry (ESI** -**MS):** Positive-ion ESI(+) mass spectra were obtained either on a Bruker Esquire2000 Ion Trap Spectrometer or a Bruker MicroTOF Spectrometer. Samples were prepared in either 100 % H_2_O or 95 % MeOH/5 % H_2_O mixture and typically injected at 2 μL min^–1^, nebulizer gas (N_2_) 25 psi, dry gas (N_2_) 9 L min^–1^, dry temp. 300 °C, capillary –4000 V (+ mode), end plate offset –500 V, capillary exit 70–170 V, and Oct RF 50–400 V pp, unless otherwise stated. Data were processed using DataAnalysis version 3.3 (Bruker Daltonics).

**UV/Vis Absorption Spectroscopy:** UV/Vis absorption spectra were recorded on a Cary 50-Bio spectrophotometer using 1-cm path length quartz cuvettes (600 μL) and a PTP1 Peltier temperature controller. Spectra were recorded at 310 K in deionised water from 200 to 800 nm and were processed using UV-Winlab software for Microsoft Windows 95®.

**Aqueous Solution Chemistry:** Hydrolysis of the Ru^II^ arene complexes was monitored by UV/Vis spectroscopy. The nature of the hydrolysis products as well as the extent of the reactions were verified by ^1^H NMR spectroscopy or ESI-MS. For UV/Vis spectroscopy, the complexes were dissolved in methanol and diluted with H_2_O to give 100 μm solutions (5 % MeOH/95 % H_2_O). The absorbance was recorded at several time intervals at the selected wavelength (at which the maximum changes in absorbance were registered) over ca. 4 h at 310 K. The data were then subjected to kinetic analysis for the first and second aquation steps. Plots of the change in absorbance with time were computer-fitted to the appropriate biexponential kinetic equation using Origin version 8.0 (Microcal Software Ltd.) to give the half-lives (t_1/2_, min) and rate constants (k_n_, min^–1^). For ^1^H NMR spectroscopy, the complexes were dissolved in [D_4_]MeOD and diluted with D_2_O to give 100 μm solutions (5 % [D_4_]MeOD/95 % D_2_O). The spectra were acquired at various time intervals on a Bruker DMX 700 spectrometer (^1^H = 700 MHz) using 5 mm diameter tubes. All data processing was carried out using XWIN NMR version 2.0 (Bruker U. K. Ltd.). ^1^H NMR signals were referenced to dioxane as an internal reference (*δ* = 3.71). The relative amounts of Ru^II^ arene halido species or aqua adducts were determined by integration of peaks in ^1^H NMR spectra.

**Rate of Arene Loss:** The complexes were dissolved in [D_4_]MeOD and diluted with D_2_O to give 100 μm solutions (5 % [D_4_]MeOD/95 % D_2_O). Arene loss over time was followed by ^1^H NMR spectroscopy at 310 K for ca. 4 h.

**Interactions with Nucleobases:** The reactions of 5 % [D_4_]MeOD/95 % D_2_O solutions of the Ru^II^ arene complexes (100 μm) with 2 mol equiv. of 9-ethylguanine (9-EtG) were monitored over time. ^1^H NMR spectra were recorded at 310 K at various time intervals for 24 h.

**Cancer Cell Growth Inhibition:** After plating, human ovarian A2780 cancer cells were treated with Ru^II^ arene complexes on day 3 at concentrations ranging from 0.1 to 100 μm. Solutions of the Ru^II^ complexes were made up in 0.125 % DMSO to assist dissolution (0.03 % final concentration of DMSO per well in the 96-well plate). Cells were exposed to the complexes for 24 h, washed, supplied with fresh medium, allowed to grow for three doubling times (72 h), and then the protein content measured (proportional to cell survival) using the sulforhodamine B (SRB) assay.^51^

**Supporting Information** (see footnote on the first page of this article): Experimental data for the X-ray crystal structures of complexes **1a** and **2**, Tables S1–S3. Mass-to-charge ratios obtained from ESI-MS spectra for the products of hydrolysis and from nucleobase interactions, Tables S4 and S5. IC_50_ values, Table S6. ^1^H NMR spectrum of **1** in [D_6_]acetone solution (Figure S1). Intermolecular π-π stacking of the benzyl rings of two *N*,*N*-dimethylbenzylammonium cations in the crystal structure of [(η^6^-*p*-cym)Ru(NH_3_)Cl_2_]**·**(dmba–H)(PF_6_) (**1a**), Figure S2. Kinetic fits for the aquation reactions of complexes **1** and **2** (Figure S3). ^1^H NMR spectra of complex **1** recorded at different stages of aquation (Figure S4). Time-dependence ^1^H NMR spectra of complexes **1** and **2** in the presence of 9-EtG (Figure S5).

## Supplementary material

Detailed facts of importance to specialist readers are published as ”Supporting Information”. Such documents are peer-reviewed, but not copy-edited or typeset. They are made available as submitted by the authors.
