# Supplementary material for: Organometallic cis-Dichlorido Ruthenium(II) Ammine Complexes
Source: Eur J Inorg Chem. 2011 Jun 22;2011(21):3257–64. doi: 10.1002/ejic.201100250 (PMC3744359; doi:10.1002/ejic.201100250)
Supplement: Supplementary file 1 [file ejic2011-3257-SD1.pdf]

Eur. J. Inorg. Chem. **2011** · © WILEY-VCH Verlag GmbH & Co. KGaA, 69451 Weinheim, 2011 · ISSN 1434–1948

SUPPORTING INFORMATION

DOI: 10.1002/ejic.201100250
Title: Organometallic *cis*-Dichlorido Ruthenium(II) Ammine Complexes
Author(s): Soledad Betanzos-Lara, Abraha Habtemariam, Guy J. Clarkson, Peter J. Sadler*

Table S1. Crystallographic data for [(η^6-*p*-cym)Ru(NH_3)Cl_2]·[(dmba–H)(PF_6)] (**1a**) and [η^6-bip)Ru(NH_3)Cl_2] (**2**).

	(**1a**)	(**2**)
Formula	$C_{19}H_{31}Cl_2F_6N_2PRu$	$C_{12}H_{13}Cl_2NRu$
Molar Mass	604.40	343.20
Crystal system	Yellow needle	Orange block.
Crystal size/mm^3	0.16 × 0.02 × 0.02	0.10 × 0.06 × 0.02
Space group	P-1	P-1
Crystal system	Triclinic	Triclinic
a/ Å	8.8456(7)	5.7821(3)
b/ Å	10.9737(9)	8.9621(4)
c/ Å	13.4054(12)	11.9292(6)
α/deg	110.456(4)	93.831(3)
β/deg	91.907(5)	101.256(2)
γ/deg	90.991(6)	105.661(3)
Z	2	2
R [F>4σ (F)]	0.0884	0.0353
Rw	0.1866	0.0664
GOF	1.123	1.060
Δρ max and min/eÅ^{-3}	1.102, –0.886	0.599, –0.687

Table S2. Hydrogen bond lengths (Å) and angles (°) in the X-ray crystal structure of [η^6-*p*-cym)Ru(NH_3)Cl_2]·[(dmba–H)(PF_6)] (**1a**).

D	H	A	D–H (Å)	H···A (Å)	D···A (Å)	D–H–A (°)
N(208)	H(28A)	Cl(1)	0.85(2)	2.78(8)	3.407(9)	132(9)
N(208)	H(28A)	Cl(2)	0.85(2)	2.54(7)	3.279(9)	145(10)
N(1)	H(1A)	Cl(1)	0.85(2)	2.56(3)	3.392(9)	167(9)[a]
N(1)	H(1C)	F(14)	0.85(2)	2.40(6)	3.142(9)	147(9)[a]
N(1)	H(1B)	Cl(2)	0.85(2)	2.63(3)	3.460(9)	165(9)[b]
C(110)	H(11B)	Cl(2)	0.98	2.67	3.626 (10)	165.6[b]
C(110)	H(11A)	F(11)	0.98	2.43	3.351(11)	157.0[c]
C(110)	H(11A)	F(15)	0.98	2.67	3.430(11)	137.2[c]

Symmetry operators used to generate equivalent atoms involved in these contacts:
[a] [–x+1, –y+1, –z+1]
[b] [–x, –y+1, –z+1]
[c] [1–x, –y, 1–z].

Table S3. Hydrogen bond lengths (Å) and angles (°) in the X-ray crystal structure of [η^6-bip)Ru(NH_3)Cl_2] (**2**).

D	H	A	D–H (Å)	H···A (Å)	D···A (Å)	D–H–A (°)
N(1)	H(1B)	Cl(1)	0.910	2.667	3.410	139.42[a]
N(1)	H(1C)	Cl(1)	0.910	2.742	3.587	155.02[b]
N(1)	H(1D)	Cl(2)	0.910	2.606	3.388	144.41[c]

Symmetry operators used to generate equivalent atoms involved in these contacts:
[a] [–x+1, –y, –z+1]
[b] [x–1, y, z]
[c] [–x+1, –y, –z+1]

Table S4. Mass-to-charge ratios obtained from ESI-MS spectra for the products of hydrolysis of Ru^{II} arene complexes **1** and **2.**

	Observed peak $[M]^+$	Chemical formula Calc *m*/*z*	Found *m*/*z*
(**1**)	{[(η^6-*p*-cym)Ru(NH_3)(H_2O)$_2$]−[(H^+)]}$^+$	$C_{10}H_{20}NO_2Ru$ 288.35	288.90
(**2**)	[{[(η^6-bip)Ru(NH_3)(H_2O)$_2$]−[(H^+)]}$^+$	$C_{12}H_{17}NO_2Ru$ 308.34	308.90

Table S5. Mass-to-charge ratios obtained from ESI-MS spectra for the products of 9-EtG interactions of Ru^{II} arene complexes **1** and **2**.

	Observed peak $[M]^{n+}$	Chemical formula Calc *m*/*z*	Found *m*/*z*
(**1**)	{[(η^6-*p*-cym)Ru(9-EtG-*N7*)(H_2O)$_2$]$^{2+}$–[H^+]}$^+$	$C_{17}H_{27}N_5O_3Ru$ 450.50	450.06
	{[(η^6-*p*-cym)Ru(9-EtG-*N7*)]$^{2+}$	$C_{17}H_{23}N_5ORu$ 207.23	207.54
(**2**)	{[(η^6-bip)Ru(9-EtG-*N7*)(H_2O)$_2$]$^{2+}$–[H^+]}$^+$	$C_{19}H_{22}N_5O_3Ru$ 469.50	469.03
	[(η^6-bip)Ru(9-EtG-*N7*)Cl]$^+$	$C_{16}H_{19}ClN_5ORu$ 469.91	470.03
	[(η^6-bip)Ru(9-EtG-*N7*)]$^{2+}$	$C_{19}H_{18}N_5ORu$ 217.23	217.53
(**1**),(**2**)	{[Ru(9-EtG-*N7*)$_2$]$^{2+}$–[H^+]}$^+$	$C_{14}H_{17}N_{10}O_2Ru$ 458.43	458.09

Table S6. IC_{50} values for Ru^{II} arene complexes **1** and **2** against the A2780 human ovarian cancer cell line.

Compound	IC_{50} μM
(**1**) $[(\eta^6\text{-}p\text{-cym})Ru(NH_3)Cl_2]$	> 100
(**2**) $[(\eta^6\text{-bip})Ru(NH_3)Cl_2]$	> 100
Cisplatin	0.5

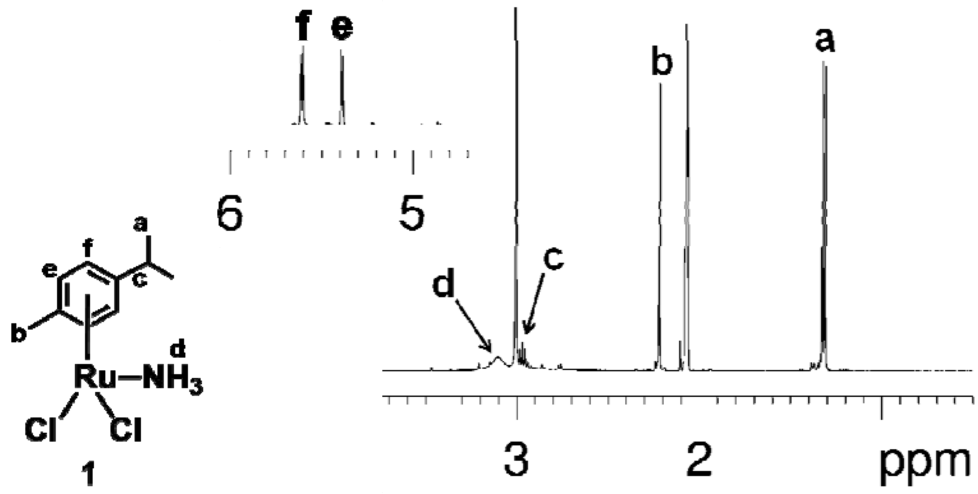

Figure S1. ^{1}H NMR spectrum of [(η^{6}-*p*-cym)Ru(NH$_3$)Cl$_2$] (**1**) in acetone-d_6 solution. The assignments of the peaks are indicated on the structure.

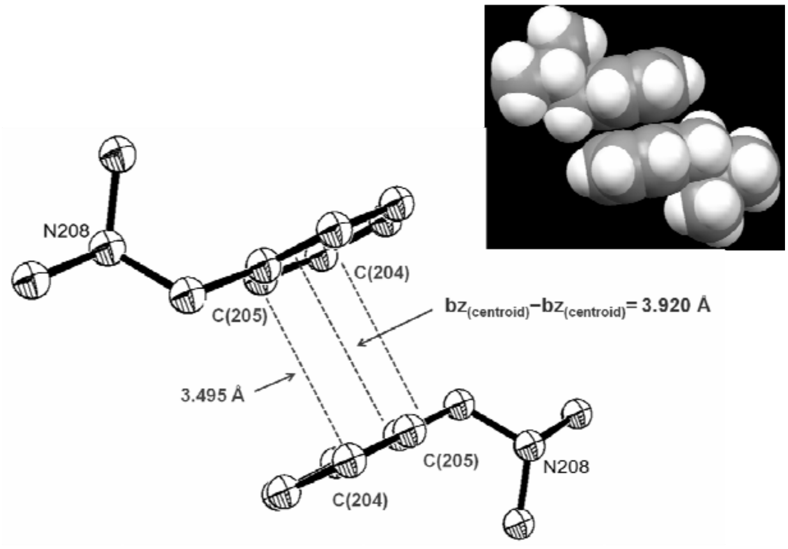

Figure S2. Intermolecular π-π stacking of the benzyl rings of two N,N-dimethylbenzylammonium cations in the crystal structure of [(η^6-*p*-cym)Ru(NH_3)Cl_2]·(dmba–H)(PF_6) (**1a**). Inset: space-filling model.

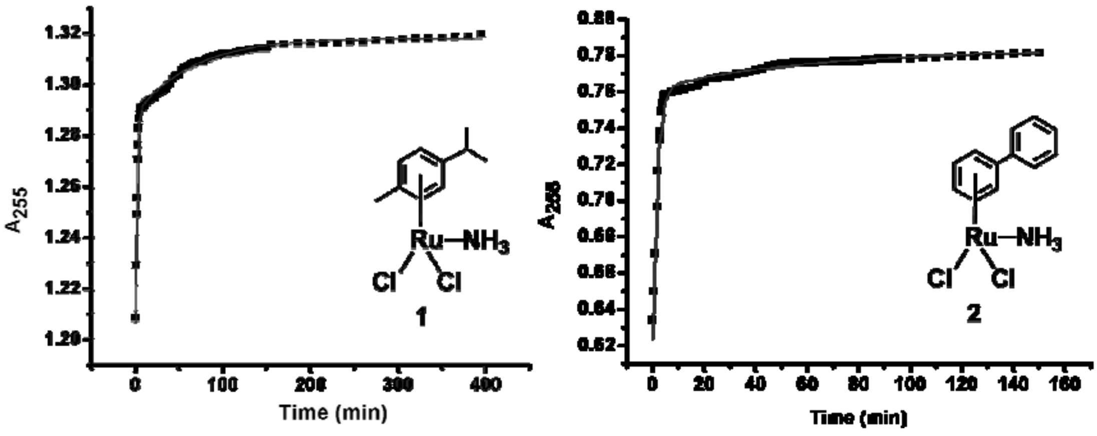

Figure S3. Dependence of the absorbance at 255 nm on time over *ca.* 7 h during aquation of [(η^6-*p*-cym)Ru(NH_3)Cl_2] (**1**) or [(η^6-bip)Ru(NH_3)Cl_2] (**2**) (100 μM in 5% MeOH/95% H_2O) at 310 K.

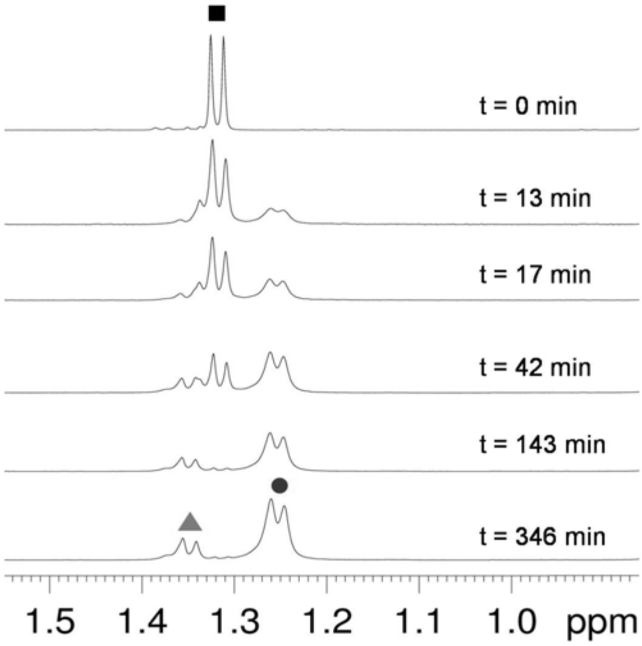

Figure S4. The aliphatic region of the ^{1}H NMR spectrum of [(η^6-*p*-cym)Ru(NH_3)Cl_2] (**1**) in 5% MeOD-d_4/95% D_2O at 310 K recorded at different stages of aquation.
■ = [(η^6-*p*-cym)Ru(NH_3)$(Cl)_2$], ▲ = [(η^6-*p*-cym)Ru(NH_3)(OH_2)Cl]$^+$,
● = [(η^6-*p*-cym)Ru(NH_3)$(OH_2)_2$]$^{2+}$.

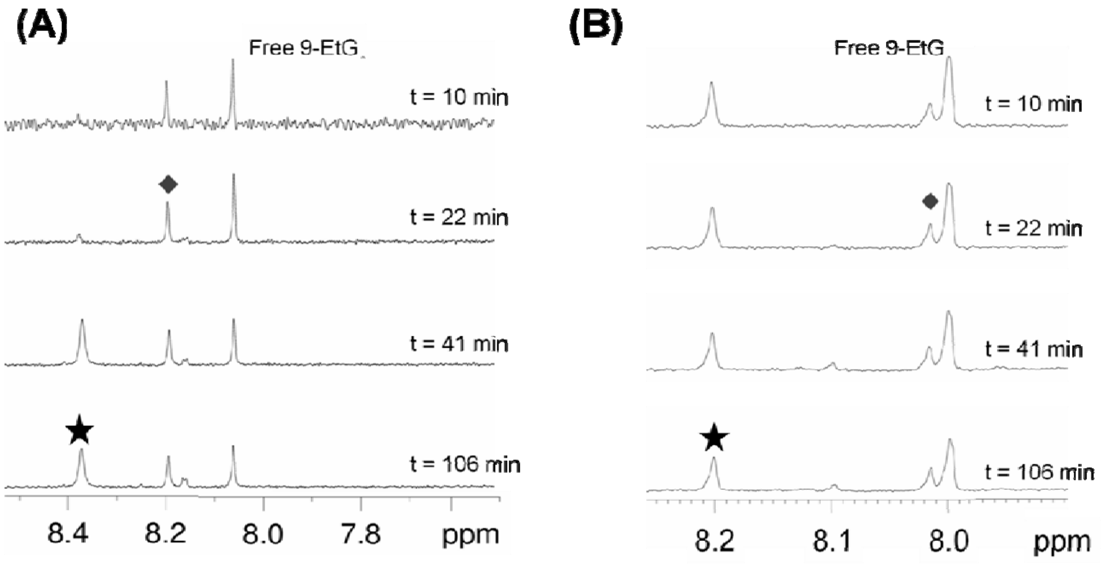

Figure S5. Time dependence of the aromatic region of ^{1}H NMR spectra of (A) [(η^6-*p*-cym)Ru(NH_3)Cl_2] (**1**) and (B) [(η^6-bip)Ru(NH_3)Cl_2] (**2**) in 5% MeOD-d_4/95% D_2O at 310 K in the presence of 2 mol equiv of 9-EtG. ♦ = [(η^6-*p*-cym)Ru(NH_3)(9-EtG-*N7*)Cl]$^+$ (**1-EtG**)/[(η^6-bip)Ru(NH_3)(9-EtG-*N7*)Cl]$^+$ (**2-EtG**); ★ = [(η^6-*p*-cym)Ru(NH_3)(9-EtG-*N7*)$_2$]$^{2+}$ (**1-EtG$_2$**)/[(η^6-bip)Ru(NH_3)(9-EtG-*N7*)$_2$]$^{2+}$ (**2-EtG$_2$**).
